# A Case of Epstein-Barr Virus–Associated Transplanted Kidney Post-Transplant Lymphoproliferative Disorder Complicated by Hemophagocytic Lymphohistiocytosis, Six Years after Transplantation

**DOI:** 10.70352/scrj.cr.25-0669

**Published:** 2026-03-06

**Authors:** Takato Waseda, Naohiro Aida, Taihei Ito, Takashi Kenmochi

**Affiliations:** Department of Transplantation and Regenerative Medicine, School of Medicine, Fujita Health University, Toyoake, Aichi, Japan

**Keywords:** post-transplant lymphoproliferative disease, Epstein–Barr virus, living-donor kidney transplantation, thrombotic microangiopathy, hemophagocytic lymphohistiocytosis

## Abstract

**INTRODUCTION:**

We present a case of acute kidney failure due to post-transplant lymphoproliferative disorder (PTLD) in the transplanted kidney 6 years after transplantation. Epstein–Barr virus (EBV) infection was identified as the likely trigger, and reducing immunosuppression was sufficient to resolve both inflammation and renal failure.

**CASE PRESENTATION:**

A female patient in her twenties with acute renal failure had undergone a living-donor kidney transplantation from an EBV-positive donor, who was her mother, 6 years earlier and continued to receive immunosuppressive medication, including tacrolimus, everolimus, mycophenolate mofetil, and methylprednisolone. The patient was negative for EBV antibodies before transplantation but had become positive after 6 months, despite no detectable EBV DNA in her blood. Her creatinine (CRE) level had remained stable at around 1.5 mg/dL, but suddenly rose to 2.0 mg/dL at 6 years after transplantation. Graft rejection was suspected, and steroid pulse treatment was performed, but this did not improve renal function, while fever, thrombocytopenia, and hemolytic anemia appeared. Thrombotic microangiopathy (TMA) was suspected, so tacrolimus and everolimus were discontinued and mycophenolate mofetil was increased. However, transplant kidney biopsy showed no findings of TMA. Therefore, these findings were suspected to be symptoms of hemophagocytic lymphohistiocytosis (HLH). PCR testing revealed EBV viremia, and the patient was diagnosed with EBV-associated HLH. CRE rose to 6.0 mg/dL but gradually decreased after changing immunosuppressive drugs, and the patient’s fever resolved. Staining of the kidney biopsy with Epstein–Barr virus encoded RNA (EBER) was positive for infiltrated lymphocytes, leading to the diagnosis of PTLD. No other lesions, such as enlarged lymph nodes, were observed on imaging. Since her symptoms resolved, she has been under outpatient observation with mycophenolate mofetil and methylprednisolone without additional treatment. Her blood EBV nucleic acid level did not increase, and CRE has remained stable at 1.6 mg/dL.

**CONCLUSIONS:**

In a rare case, HLH developed during immunosuppressive treatment of acute renal failure suspected to represent graft rejection. It then was diagnosed as EBV-induced late PTLD instead, and the patient’s condition improved and stabilized after immunosuppressant regimen adjustment. We recommend regular EBV-monitoring and consideration of the possibility of graft PTLD in similar cases.

## Abbreviations

CMV
: cytomegalovirus
CNI
: calcineurin inhibitor
CRE
: creatinine
EBER
: Epstein–Barr virus–encoded RNA
EBV
: Epstein–Barr virus
EVL
: everolimus
FK
: tacrolimus
HLH
: hemophagocytic lymphohistiocytosis
LPD
: lymphoproliferative disorder
MMF
: mycophenolate mofetil
mPSL
: methylprednisolone
PTLD
: post-transplant lymphoproliferative disease
RIS
: reduction in immunosuppression
TMA
: thrombotic microangiopathy

## INTRODUCTION

PTLD is one of the most serious complications after transplantation.^1–3)^ PTLD is classified into early and late onset forms.^1,4)^ Early-onset PTLD occurs within 1 year after transplantation, while late-onset PTLD occurs after 1 year.^1)^ The risk factors for the development of early-onset and late-onset PTLD are different. Risk factors for early-onset PTLD include preoperative EBV seronegativity, polyclonal anti-lymphocyte antibodies, and young age (infancy and early childhood).^1)^ On the other hand, late-onset PTLD is associated with a period of immunosuppression and older age (adulthood).^1)^ EBV is strongly implicated in the pathogenesis of PTLD, which is more likely to occur early after transplantation if preoperatively the patient is seronegative for EBV antibodies,^5,6)^ especially if the donor has been infected with EBV.^1,7)^ Therefore, if the recipient is preoperatively EBV-negative and the donor was previously infected, it is very important to follow-up with periodic EBV monitoring and imaging evaluation starting from early after transplantation.

Our case report presents a case of acute renal failure that eventually was diagnosed as EBV-induced PTLD localized to the transplanted kidney 6 years after transplantation. Because we first suspected kidney graft rejection, steroid pulse treatment was initiated for immunosuppression, but the situation only worsened with the patient developing HLH. After realizing that this was a case of virus infection instead, RIS therapy was initiated and the patient’s inflammation subsided and her renal condition improved.

## CASE PRESENTATION

A female patient in her twenties had been treated with a living donor kidney transplantation donated from her mother 6 years prior, because she presented with chronic renal failure due to autosomal recessive polycystic kidney disease. Her ongoing transplant maintenance immunotherapy included FK, EVL, MMF, and mPSL. Before transplantation, EBV IgG of the recipient had been negative, while that of the donor was positive. Six months after transplantation, the patient tested positive for anti-EBV antibodies despite remaining serum EBV-DNA negative, and she was subjected to regular follow-up examinations. She developed CMV infection 1 month after transplantation and required a total of 4 hospitalizations due to CMV infection within the first year. The patient’s CRE remained stable around 1.5 mg/dL, but 6 years after transplantation, it rose sharply to 2.0 mg/dL, and the patient was hospitalized for close examination. On the day of admission, the patient was oriented, and her vital signs were as follows: temperature 37.1°C; oxygen saturation 96% on room air; blood pressure 132/83 mmHg; and pulse rate 78 beats/minute. Blood and urine laboratory data at admission showed (**Table 1**) mild anemia (Hb 9.4 g/dL) and renal function impairment (CRE 2.06 mg/dL); no electrolyte abnormalities (sodium, 141 mEq/L; potassium, 3.6 mEq/L; chloride, 106 mEq/L; calcium, 9.5 mEq/L); and urine protein semi-quantitative (2+) and urinary occult blood reactions (2+).

**Table 1:** Laboratory findings on admission and on day 14

On admission		On day 14	
Complete blood cell counts			
WBC	11.5 × 10^3^/μL	WBC	11.8 × 10^3^/μL
RBC	3.84 × 10^6^/μL	RBC	3.30 × 10^6^/μL
Hb	9.4 g/dL	Hb	8.2 g/dL
Hct	30.5%	Hct	26.3%
PLT	31.5 × 10^4^/μL	PLT	12.4 × 10^4^/μL
MCV	79 fL	MCV	80 fL
		Schistocytes	(+)
		Haptoglobin	3.0 mg/dl
Blood biochemistry			
TP	7.7 g/dL	TP	6.8 g/dL
Alb	3.9 g/dL	Alb	3.1 g/dL
AST	26 U/L	AST	48 U/L
ALT	31 U/L	ALT	31 U/L
LDH	269 U/L	LDH	826 U/L
ALP	87 U/L	ALP	105 U/L
BUN	56.8 mg/dL	BUN	70.3 mg/dL
CRE	2.06 mg/dL	CRE	4.10 mg/dL
Cys-C	3.81 mg/L	Cys-C	7.90 mg/L
CRP	0.08 mg/dL	CRP	2.79 mg/dL
Na	141 mEq/L	Na	132 mEq/L
K	3.6 mEq/L	K	3.7 mEq/L
Cl	106 mEq/L	Cl	96 mEq/L
Ca	9.5 mEq/L	Ca	8.9 mEq/L
FK trough	7.8 ng/mL	Ferritin	9929 ng/mL
		sIL-2R	1869 U/mL
Urine biochemistry			
Urine protein semi-quantitative	(2+)	Urine protein semi-quantitative	(2+)
Urinary occult blood reaction	(2+)	Urinary occult blood reaction	(2+)

**Fig. 1:**
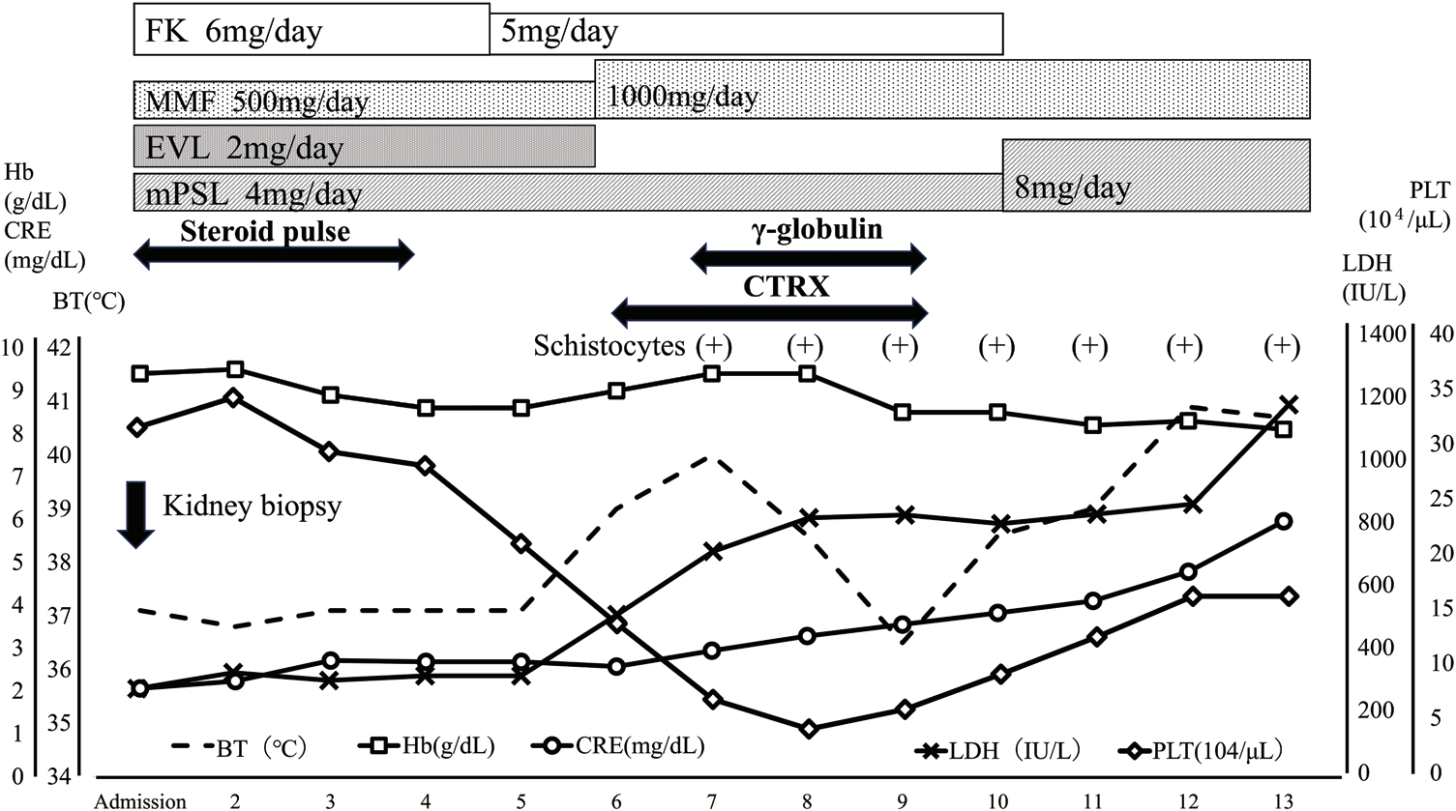
Admission progress chart from admission to day 13. A steroid pulse was administered, but creatinine level gradually rose. Post-treatment fever, thrombocytopenia, and elevated LDH were observed. BT, body temperature; CTRX, ceftriaxone; EVL, everolimus; FK, tacrolimus; LDH, lactate dehydrogenase; MMF, mycophenolate mofetil; mPSL, methylprednisolone; PLT, platelet

**Fig. 2:**
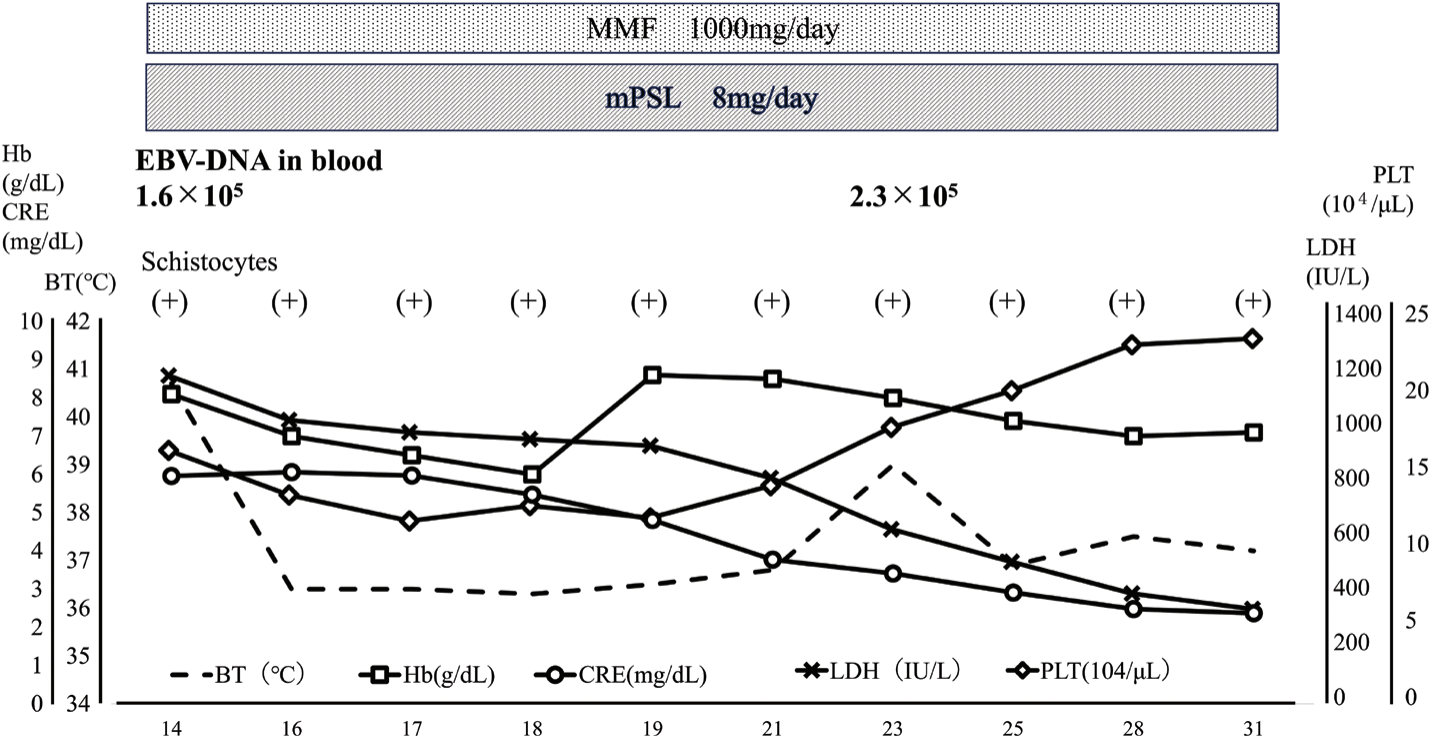
Hospitalization progress chart from the 14th day of admission to discharge. After diagnosing EBV-PTLD and HLH, the patient’s condition improved spontaneously with only a reduction in immunosuppressive. BT, body temperature; EBV, Epstein–Barr virus; HLH, hemophagocytic lymphohistiocytosis; LDH, lactate dehydrogenase; MMF, mycophenolate mofetil; mPSL, methylprednisolone; PLT, platelet; PTLD, post-transplant lymphoproliferative disease

**Fig. 3:**
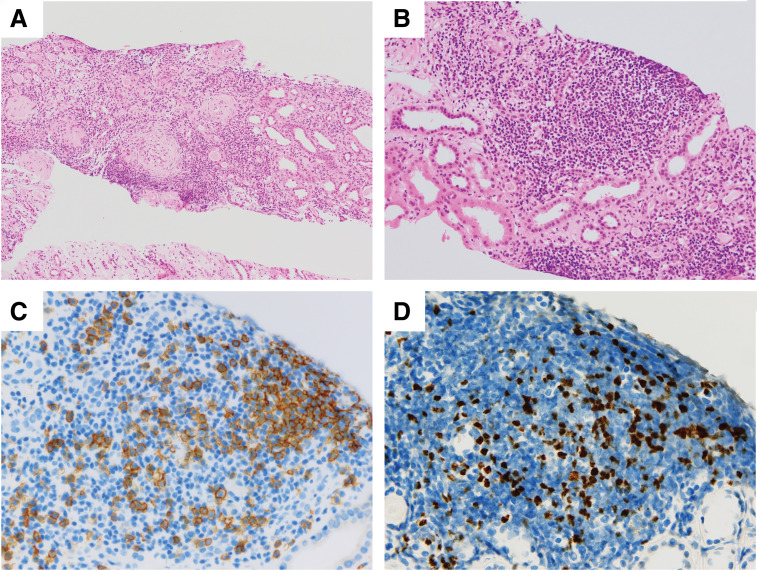
Pathology findings in renal biopsy specimens. (**A**) H&E staining, ×100. (**B**) H&E staining, ×200. There was no evidence of tubulitis. Lymphocytic infiltration and fibrosis were observed in the interstitium. Sclerotic changes were observed in half of the glomeruli. (**C**) Immunostaining for CD20 showing positive cells, ×400. Most of the aggregated lymphocytes were CD3-positive cells, but a significant number of CD20-positive cells were also observed. (**D**) EBER staining showing positive cells, ×400. CD-20-positive cells were EBER-positive, suggesting EBV infection. EBER, Epstein–Barr virus encoded RNA; EBV, Epstein–Barr virus; H&E, hematoxylin and eosin

**Fig. 4:**
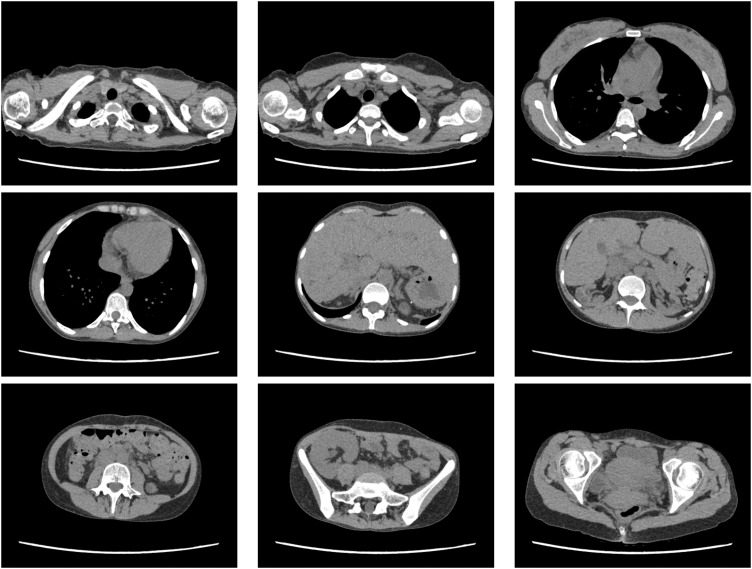
Chest and abdominal CT images showed no mass lesions other than in the transplanted kidney. No neoplastic lesions were identified other than the transplanted kidney.

Late acute graft rejection was clinically suspected; thus, a renal biopsy was performed immediately after admission, and steroid pulse therapy was initiated (**Fig. 1**). Despite a steroid pulse for 3 days, the CRE rose to 3.0 mg/dL on the 4th day of admission. On the 5th day of hospitalization, the patient developed a fever exceeding 39°C. Bacterial infection such as pyelonephritis or catheter-related infection was suspected and antibiotics (ceftriaxone) and γ-globulin (daily 5000 mg Kenketsu Venilon-I by intravenous injection for 3 days) were administered. The fever persisted, while also progressive thrombocytopenia (PLT dropped sharply from 2.0 × 10^5^/μL to 4.0 × 10^4^/μL over 3 days), worsening anemia (Hb level was 9.4 g/dL), and the appearance of crushed erythrocytes were observed. Low levels were observed for haptoglobin (3mg/dL). Although D-dimer level was high (3.5 μg/mL), any other Coagulation Fibrinolysis Examination showed normal results (PT-INR 0.84, APTT 29.2s, fibrinogen 332 mg/dL). The ADAMTS13 activity was 44%, and the ADAMTS13 inhibitor was less than 0.5 BU/mL. High levels were observed for lactate dehydrogenase (LDH 814U/L). The renal function was worse (BUN 64.4mg/dL, CRE 3.28 mg/dL). Therefore, at first, TMA was suspected and FK and EVL were gradually discontinued. Histopathologic findings of her renal biopsy taken at admission (it takes over 3 days to make a diagnosis) revealed no evidence of tubulitis or microvascular injury, suggesting graft rejection. However, sclerotic glomeruli, tubular atrophy, cellular infiltration into the interstitium, and interstitial fibrosis were observed, suggesting the influence of CNI toxicity. Arterial lesions were not revealed, thus there was no evidence of TMA. Her renal function worsened further, and her fever didn’t resolve until more than a week later (**Fig. 2**). The presence of CNI toxicity and the suspicion of infection lead to the decision of not to reintroduce the discontinued immunosuppressants. By the 14th day of hospitalization, the patient exhibited a high fever exceeding 40°C (**Fig. 2**), but the inflammatory response was only mildly elevated with a CRP of 2.79 mg/dL (**Table 1**). The haptoglobin level was low, and crushed red blood cells were observed, indicating hemolytic anemia. Meanwhile, creatinine levels increased to 4.10 mg/dL, and high levels were observed for LDH (826 U/L), ferritin (9929 ng/mL), and sIL-2R (1869 U/mL) (**Table 1**). The blood examination results suggested hemophagocytic syndrome, leading us to conduct a comprehensive virus search. EBV-DNA in the blood was found elevated at 1.0 × 10^5^ (PCR analysis by SRL, Inc., Tokyo) and the patient was diagnosed with having EBV-associated HLH. This was consistent with subsequent immunostaining of the renal biopsy specimen, as this identified cells—within the accumulations of lymphocytes—that were positive for both CD20 (a B cell marker) and EBER (**Fig. 3C**, **3D**). To supplement the pathological findings, lymphocyte infiltration into the interstitium included EBER-positive and CD20-positive cells as well as CD79a-positive cells, but CD3-positive cells were predominant. The lambda/kappa ratio was nearly equal, indicating polyclonal proliferation. Based on these findings, the histological diagnosis was lymphoproliferative disorder in the WHO 2017 classification. PET-CT could not be performed, so plain CT was conducted. Chest and abdominal CT imaging did not reveal mass lesions other than in the transplanted kidney (**Fig. 4**), with no evidence of enlarged superficial lymph nodes or other physical findings.

Based on these results, the patient was diagnosed with EBV-induced PTLD localized to the transplanted kidney. The early reduction in RIS may have helped the patient to control the EBV infection and recover from HLH and fever. Since the response to RIS was favorable after diagnosis, a bone marrow examination was not performed. Her kidney function also recovered, because after CRE had temporarily increased to above 6.0 mg/dL around day 14 of hospitalization, it decreased to about 2.0 mg/dL after another 2 weeks (**Figs. 1**, **2**). On the 28th day after admission, no significant increase in blood EBV-DNA was observed, and the patient was discharged from the hospital. Three months after discharge, the patient did not exhibit thrombocytopenia and her CRE had remained stable at 1.6 mg/dL (**Table 2**). Since her discharge, our center continues to follow-up with the patient on an outpatient basis, basically prescribing 2 immunosuppressive drugs: MMF 1000 mg/day and mPSL 8 mg/day. Although EBV-DNA in the blood was still persistently positive for 2.8 × 10^3^ copies after 4 years, no serious complications or progression of PTLD have been observed, and her CRE is stable at 1.5 mg/dL.

**Table 2:** Laboratory findings at 3 months after discharge

3 months after discharge	
Complete blood cell counts	
WBC	10.9 × 10^3^/μL
RBC	3.59 × 10^6^/μL
Hb	10.3 g/dL
Hct	33.4%
PLT	40.6 × 10^4^/μL
Blood Biochemistry	
TP	6.9 g/dL
Alb	4.2 g/dL
AST	16 U/L
ALT	14 U/L
LDH	164 U/L
ALP	56 U/L
BUN	38.3 mg/dL
CRE	1.61 mg/dL
Cys-C	2.66 mg/L
Urine protein semi-quantitative	(2+)
Urinary occult blood reaction	(1+)
EBV-DNA	1.1 × 10^5^

EBV, Epstein–Barr virus

## DISCUSSION

This study describes a rare case of acute kidney failure induced by EBV-induced late-onset PTLD, initially misinterpreted as graft rejection. This misinterpretation led to enhanced immunosuppression treatment to protect the graft, which likely exacerbated the EBV replication and caused HLH. Both the inflammation and kidney failure improved after RIS.

In kidney transplantation, the incidence of PTLD is highest immediately after transplantation, and while this incidence decreases thereafter, it increases again from about 4 years after transplantation.^8,9)^ Approximately 10% of PTLD cases occur in the kidney graft, whereas most of these cases are early onset. The involvement of EBV in PTLD decreases in the late-onset cases, affecting approximately half of cases; therefore, the clinical presentation described in this report is rare. It is also important to note that primary graft PTLD is one of the diseases that may lead to allograft dysfunction.^3)^ Although our patient tested positive for EBV-IgG 6 months after transplantation, her serum EBV-DNA was negative. Therefore, her EBV infection was considered under control and PTLD was initially not suspected. However, in hindsight and given the EBV-positivity of her transplanted kidney biopsy at the day of admission, her sudden decrease in renal function can be considered a symptom of PTLD, and the steroid pulse therapy may have increased the viral load of EBV, exacerbating PTLD and inducing HLH. The risk of developing PTLD increases with the level of immunosuppression, and there have been previous reports on PTLD development after steroid pulse therapy.^10,11)^ Therefore, the importance of EBV-DNA monitoring before treatment initiation has been emphasized when inducing strong immunosuppression, such as by steroid pulse therapy.^12)^

The general strategy and challenge of treating EBV-induced PTLD is to restore EBV-specific cell immunity but maintain some level of immunosuppression to avoid graft rejection by allogeneic anti-graft immunity.^3)^ Therefore, PTLD treatment, pharmacotherapy, and auxiliary surgery are performed in stages based on the clinical situation and the histopathological characteristics.^1,13,14)^ The histopathological findings of PTLD are summarized in the WHO classification, which was revised to its latest edition in 2022. Treatment is selected based on histological classification, and chemotherapy or rituximab may be administered.^15,16)^ Some cases of PTLD are relieved by RIS alone.^17,18)^ The response to RIS varies depending on multiple factors, including the time of onset of PTLD,^18)^ and treatment with RIS in late-onset PTLD—as in the present study—has been reported to be less effective than in early-onset PTLD.^7)^ This result is thought to be partly due to the low incidence of EBV in late-onset PTLD. Commonly, RIS involves reducing the dose of calcineurin inhibitors and adding EVL, or the discontinuation of MMF.^19,20)^ In our hospital, discontinuation of calcineurin inhibitors has been established as the first choice, because of our positive experiences with remission of PTLD after discontinuing calcineurin inhibitors and using MMF and steroids. In the present case, FK was discontinued early during the treatment because TMA was suspected, and TMA is strongly associated with calcineurin inhibitor treatment and may occur as a complication after transplantation.^21,22)^ After discontinuing the calcineurin inhibitor FK, EVL was also considered as a potential causative agent of TMA and was discontinued as well. When the patient was eventually diagnosed with PTLD, the immunosuppressive treatment was considered as already sufficiently attenuated and maintained at the same level. In the case that resistance to RIS developed within approximately 2 weeks, rituximab administration was planned. The favorable response to RIS was likely due to the pathological findings of EBV-positive LPD. However, her serum EBV-DNA remains persistently positive, so RIS is being continued with strict follow-up.

The pathogenesis of HLH involves hypercytokinemia due to excessive activation of lymphocytes and macrophages, and this excessive release of cytokines results in bone marrow suppression and organ damage.^23)^ In the present case, the diagnosis of HLH was based on agreement with the following HLH-2004 criteria (5 or more criteria need to be fulfilled for this diagnosis): (1) Fever; (2) Cytopenias affecting ≥2 of 3 peripheral blood cell lineages; (3) Hypertriglyceridemia and/or hypofibrinogenemia; (4) Hemophagocytosis in the bone marrow or spleen or lymph node; (5) Ferritin ≥500μg/L; and (6) sIL-2 receptor ≥2400U/mL.^24,25)^

Although HLH in adults is rare compared with in children, it is important to suspect HLH at its early disease stage, because otherwise irreversible damage can accumulate. In the present case study, graft rejection, TMA, and HLH were first considered as differential diagnoses, but the renal biopsy results were negative for rejection and TMA, leading to HLH diagnosis and a comprehensive viral search. As a result, EBV-DNA positivity in the blood was detected and the patient was treated accordingly. The administration of RIS and γ-globulin in the early stage of HLH appears to have been successful, leading to rapid improvement of the patient’s condition. However, based on government and insurance policies, γ-globulin treatment had to be stopped after 3 days, and the continued improvement can mostly be ascribed to RIS. We assume that HLH was a steroid pulse-induced, exacerbated variant of the PTLD caused by EBV, as it is known that EBV activation may cause HLH.^23,25)^ To our knowledge, there have been no previous reports of this condition, which may indicate its extreme rarity.

Regular monitoring of EBV-DNA during post-transplant follow-up is very important, especially for high-risk recipients (donor IgG positive/recipient IgG negative). It is not recommended for checking at the onset of events suggestive of graft rejection, because EBV-DNA testing takes longer than histological diagnosis. When EBV-DNA is detected, prompt imaging and histological analysis are essential to accurately assess the infection status.^26)^ If EBV-DNA has not been detected or followed up solely with blood tests, it is recommended to perform readily available imaging tests such as CT before undergoing immunosuppressive therapy such as steroid pulse therapy. Additionally, as in the case, immunohistochemistry to check for graft PTLD is desirable. In Japan, insurance coverage for EBV-DNA analysis only began in April 2018 and typically extends only up to 1-year post-transplant. Since our patient received a kidney transplant in 2015, regular EBV monitoring was not feasible under insurance. This case underscores the importance of regular monitoring of EBV-DNA and the effectiveness of RIS in the case of HLH or PTLD.

### Summary of this case study

In this case, acute renal failure in a kidney transplant received 6 years earlier was initially suspected to be due to graft rejection. Consequently, steroid pulse therapy was initiated, but this appeared to worsen the condition, leading to the development of HLH. To address HLH, immunosuppression was reduced. This turned out to be an appropriate treatment for the EBV-induced PTLD, which was eventually identified as the probable underlying cause of the acute renal failure as well as of HLH. RIS ultimately resolved both inflammation and renal failure.

## CONCLUSIONS

In cases of acute failure in a transplanted kidney, EBV-induced PTLD should be considered as one of the options, even years after post-transplant and without prior signs of an active EBV infection. Regular screening for EBV can help decide to timely initiate RIS if enhanced immunosuppression, based on a mistaken suspicion of graft rejection, led to HLH. Our RIS primarily involved discontinuing calcineurin and EVL, and resulted in improved renal function and remission of HLH.

## DECLARATIONS

### Availability of data and materials

The dataset supporting the conclusions of this article is included within the article.
